# Supplementary material for: An Optimised Monophasic Faecal Extraction Method for LC-MS Analysis and Its Application in Gastrointestinal Disease
Source: Metabolites. 2022 Nov 14;12(11):1110. doi: 10.3390/metabo12111110 (PMC9698041; doi:10.3390/metabo12111110)
Supplement: Supplementary file 1 [file metabolites-12-01110-s001.zip › Supplementary Information REVISED FINAL.pdf]

Supplementary Information for

An Optimised Monophasic Faecal Extraction Method for LC-MS Analysis and Its Application in Gastrointestinal Disease

Patricia E. Kelly [1,2], H Jene Ng [3], Gillian Farrell [1], Shona McKirdy [2,3], Richard K. Russell [2,4], Richard Hansen [2,5], Zahra Rattray [1], Konstantinos Gerasimidis [2,3] and Nicholas J. W. Rattray [1,2,*]

1 Strathclyde Institute of Pharmacy and Biomedical Sciences (SIPBS), University of Strathclyde, Glasgow G4 0RE, UK
2 Bacteria, Immunology, Nutrition, Gastroenterology and Omics (BINGO) Group, University of Glasgow, Glasgow G12 8QQ, UK
3 School of Medicine, Dentistry & Nursing, University of Glasgow, Glasgow Royal Infirmary, Glasgow G12 8QQ, UK
4 Royal Hospital for Children and Young People, 50 Little France Crescent, Edinburgh EH16 4TJ, UK
5 Royal Hospital for Children, 1345 Govan Road, Glasgow G52 4TF, UK
* Correspondence: nicholas.rattray@strath.ac.uk

Figure and Table legends in Supplementary Information

Figure S1. Differential analysis of sample weight showing volcano plot of altered metabolites, plotted as log2 fold change vs -log10P. Metabolites that are significantly increased in 50 mg samples compared to 20 mg samples are highlighted in red and those that are significantly decreased are shown in green. Differences in metabolite level were defined by a log2 fold change of 1 and the significance level was set at $p < 0.05$.

Figure S2. Metabolite class analysis of sample size. (a) Comparison of the total number of metabolites identified by chemical class in 20 mg and 50 mg samples (n=3), performed in triplicate. (b) Radar plot comparing the relative abundance of metabolite classes in 20 mg and 50 mg samples. Data were expressed as mean + SEM and statistical significance was assessed using unpaired t-test.

Figure S3. Differential analysis of extraction solvent showing volcano plot of altered metabolites between (a) MeOH vs. MeOH/ H_2O, (b) $CHCl_3$/ MeOH vs. MeOH/ H_2O, and (c) $CHCl_3$/ MeOH vs MeOH, plotted as log2 fold change vs -log10P. Metabolites that are significantly increased are highlighted in red and those that are significantly decreased are shown in green. Differences in metabolite level were defined by a log2 fold change of 1 and the significance level was set at $p < 0.05$.

Figure S4. Metabolite class analysis of extraction solvent. (a) Comparison of the total number of metabolites identified by chemical class in samples extracted with MeOH, MeOH/H_2O, and $CHCl_3$/ MeOH (n=3), performed in triplicate. (b) Radar plot comparing the relative abundance of metabolite classes in samples extracted with MeOH/ H_2O, MeOH, and $CHCl_3$/ MeOH. Data were expressed as mean + SEM and statistical significance was assessed using one-way ANOVA. *$p < 0.05$, **** $p < 0.0001$.

Figure S5. Differential analysis of extraction solvent showing the volcano plot of altered metabolites between (a) sonication vs. bead beating, (b) freeze-thaw vs. bead beating and (c) freeze-thaw vs. sonication, plotted as log2 fold change vs -log10P. Metabolites that are significantly increased are highlighted in red and those that are significantly decreased are shown in green. Differences in metabolite levels were defined by a log2 fold change of 1 and the significance level was set at $p < 0.05$.

Figure S6. Metabolite class analysis of cellular disruption method. (a) Comparison of the total number of metabolites identified by chemical class in samples extracted using bead beating, sonication, and freeze-thaw cycles (n=3), performed in triplicate. (b) Radar plot comparing the relative abundance of metabolite classes in samples extracted using bead beating, sonication, and freeze-thaw cycles. Data are expressed as mean ± SEM and statistical significance was assessed using a one-way ANOVA, **** $p < 0.0001$.

Figure S7. Differential analysis of sample-solvent ratio showing volcano plot of altered metabolites between (a) 1:10 vs. 1:5, (b) 1:20 vs. 1:5 and (c) 1:20: vs. 1:10, plotted as log2 fold change vs -log10P. Metabolites that are significantly increased are highlighted red and those that are significantly decreased are shown in green. Differences in metabolite level were defined by a log2 fold change of 1 and the significance level was set at $p < 0.05$.

Figure S8. Metabolite class analysis of sample-to-solvent ratio. (a) Comparison of the total number of metabolites identified by chemical class in samples extracted using sample-to-solvent ratios of 1:5, 1:10, 1:20 (n=3), performed in triplicate. (b) Radar plot comparing the relative abundance of metabolite classes in samples extracted using 1:5, 1:10, 1:20. Data were expressed as mean ± SEM and statistical significance was assessed using a one-way ANOVA., ** $p < 0.01$, **** $p < 0.0001$.

Figure S9. Comparison of individual optimization experiments. (a) Total number of m/z features and (b) total number of identified metabolites given by optimal parameters of each experiment. Data were expressed as mean ± SEM and statistical significance was assessed using a one-way ANOVA. **$p < 0.01$, *** $p < 0.001$, **** $p < 0.0001$.

Figure S10. Differential analysis of cell lysis techniques. Volcano plot of (a) HC vs. CD; (b) CoD vs. CD (c) H vs. CoD, for all patients. Log2 fold change vs. -log10P. Metabolites. Metabolites that are significantly increased are highlighted in red and those that are significantly decreased are shown in green. Differences in metabolite level were defined by a log2 fold change of 1 and the significance level was set at $p < 0.05$.

Figure S11. Central network analysis of developed metabolite extraction method. Circles shown in green represent metabolites successfully extracted using the developed method and circles shown in red represent metabolites not found using the developed method. C1P, Ceramide-1-phosphate; AA, Arachidonic acid; EPA, Eicosapentanenoic acid; DGLA, Dihomo-gamma linolenic acid.

Figure S12. Summary of the developed methodology pipeline. Multi-parameter analysis showed that 50 mg samples give the strongest MS output, and from the extraction solvents analysed, MeOH is the most effective. Additionally, cellular metabolite release is optimal using bead beating as the cell lysis method. Combining optimised parameters provides an experimental protocol for faecal metabolite extraction that can be used for metabolomic analysis.

Table S1. Untargeted metabolomics experiment elution gradient. Mobile phase A, 99.9% water + 0.1% formic acid; Mobile B, 99.99% MeOH + 0.1% formic acid.

Table S2. Targeted metabolomics experiment elution gradient. Mobile phase A, 99.9% H_2O + 0.1% formic acid; Mobile phase B, 99.9% Acetonitrile + 0.1% formic acid.

Table S3. Overview of Untargeted Metabolite Identification Levels.

Table S4. List of metabolites included in targeted metabolomics method.

Table S5. Parameters of Compound Discoverer workflow.

Table S1. Untargeted metabolomics experiment elution gradient. Mobile phase A, 99.9% water + 0.1% formic acid; Mobile B, 99.99% MeOH + 0.1% formic acid.

Time (min)	Mobile Phase A (%)	Mobile Phase B (%)	Flow rate (mL/min)
0.0	99.0	1.0	0.4
0.5	99.0	1.0	0.4
2.0	50.0	50.0	0.4
10.5	1.0	99.0	0.4
11.0	1.0	99.0	0.4
11.5	99.0	1.0	0.4
14.9	99.0	1.0	0.4
15.0	99.0	1.0	0.4

Table S2. Targeted metabolomics experiment elution gradient. Mobile phase A, 99.9% H_2O + 0.1% formic acid; Mobile phase B, 99.9% Acetonitrile + 0.1% formic acid.

Time (min)	Mobile Phase A (%)	Mobile Phase B (%)	Flow rate (mL/min)
0	100	0	0.4
2	100	0	0.4
5	75	25	0.4
11	65	35	0.4
15	5	95	0.4
20	5	95	0.4
20.1	100	0	0.4
20.5	100	0	0.4

Table S3. Overview of Untargeted Metabolite Identification Levels

	Number of Metabolites
MSI Identification Level 2	424
MSI Identification Level 3	267

Table S4. List of metabolites included in targeted metabolomics method.

Name	Molecular Formula	Classification	Precursor m/z	Product m/z	Retention Time	Ref.(1) Precursor m/z	Ref.(1) Product m/z	Target Q1 Pre Bias	Target Collision Energy	Target Q3 Pre Bias	Ref.(1) Q1 Pre Bias	Ref.(1) Collision Energy	Ref.(1) Q3 Pre Bias
2-Aminobutyric acid	$C_4H_9NO_2$	Organic acid	104.1	58.05	2.831	104.1	41.05	-26	-12	-11	-26	-26	-17
2-Ketoglutaric acid	C_5H_6O5	Organic acid	144.9	101.1	2.317	144.9	57.05	23	12	18	23	13	21
4-Aminobutyric acid	$C_4H_9NO_2$	Organic acid	104.1	87.05	3.69	104.1	45.1	-28	-14	-17	-28	-22	-18
4-Hydroxyproline	$C_5H_9NO_3$	Amino acid	132.1	86.05	1.991	132.1	68.05	-10	-15	-18	-10	-22	-13
Acetylcarnitine	$C_9H_{17}NO_4$	Peptide	204.1	85.05	8.929	204.1	60.1	-16	-22	-18	-16	-16	-12
Acetylcholine	$C_7H_{16}NO_2$	Lipid	147.1	87.05	9.165	147.1	88.05	-12	-16	-17	-12	-16	-17
Aconitic acid	$C_6H_6O_6$	Choline	172.9	85.05	3.536	172.9	129.1	14	14	16	14	13	12
Adenine	$C_5H_5N_5$	Organic acid	136	119.05	6.46	136	65	-10	-26	-23	-10	-41	-13
Adenosine	$C_{10}H_{13}N_5O_4$	Purine base	268.1	136.05	6.764	268.1	119	-21	-18	-15	-21	-47	-23
Adenosine 3',5'-cyclic monophosphate	$C_{10}H_{12}N_5O_6P$	Nucleoside	330	136.05	6.179	330	119.1	-26	-26	-30	-26	-54	-23
Adenosine monophosphate	$C_{10}H_{14}N_5O_7P$	Nucleotide	348	136.05	2.969	348	97.1	-13	-20	-28	-13	-31	-20
Adenylsuccinic acid	$C_{14}H_{18}N_5O_{11}P$	Nucleotide	464.1	252.1	6.183	464.1	162	-18	-21	-18	-18	-47	-17
Alanine	$C_3H_7NO_2$	Organic acid	157	97.1	1.927	157	42.05	-22	-12	-18			
Allantoin	$C_4H_6N_4O_3$	Amino acid	175.1	70.1	3.365	175.1	60.1	18	15	18	18	10	15

Arginine	$C_6H_{14}N_4O_2$	Purine derivative	291	70.1	3.057	291	116.05	-13	-23	-13	-13	-16	-12
Argininosuccinic acid	$C_{10}H_{18}N_4O_6$	Amino acid	133.1	87.15	1.953	133.1	28.05	-24	-35	-14	-24	-21	-25
Asparagine	$C_4H_8N_2O_3$	Organic acid	134	74.05	1.953	134	88.1	-20	-12	-18	-20	-29	-30
Aspartic acid	$C_4H_7NO_4$	Amino acid	134	74.05	1.953	134	88.1	-30	-15	-14	-30	-13	-17
Asymmetric dimethylarginine	$C_8H_{18}N_4O_2$	Amino acid	203.1	70.1	7.207	203.1	46.1	-17	-25	-13	-17	-17	-19
Carnitine	$C_7H_{16}NO_3$	Amino acid	162.1	103.05	5.284	162.1	60.1	-13	-18	-22	-13	-17	-12
Carnosine	$C_9H_{14}N_4O_3$	Amino acid derivative	227.1	110.05	5.365	227.1	156.05	-18	-24	-23	-18	-16	-17
Cholic acid	$C_{24}H_{40}O_5$	Peptide	407.2	343.15	14.051	407.2	345.25	13	34	24	13	32	24
Choline	$C_5H_{14}NO$	Organic acid	104.1	60.05	4.436	104.1	45.1	-27	-22	-11	-27	-23	-18
Citicoline	$C_{14}H_{26}N_4O_{11}P_2$	Choline	489.1	184.1	2.045	489.1	264.05	-20	-43	-20	-20	-25	-30
Citric acid	$C_6H_8O_7$	Nucleotide	191.2	111.1	3.209	191.2	87.05	12	13	21	12	20	16
Citrulline	$C_6H_{13}N_3O_3$	Organic acid	176.1	70.05	2.321	176.1	159.05	-12	-25	-14	-12	-14	-18
Creatine	$C_4H_9N_3O_2$	Amino acid	132.1	44.05	3.431	132.1	90.05	-11	-22	-18	-11	-15	-18
Creatinine	$C_4H_7N_3O$	Organic acid	114.1	44.05	4.82			-10	-19	-18			

Cystathionine	$C_7H_{14}N_2O_4S$	Lactam	223	88.05	2.028	223	134	-17	-27	-18	-17	-15	-15
Cysteamine	C_2H_7NS	Amino acid	78.1	61.05	3.98			-19	-13	-25			
Cysteine	$C_3H_7NO_2S$	Aminothiol	122	76.05	2.148	122	59	-29	-16	-16	-29	-25	-23
Cystine	$C_6H_{12}N_2O_4S_2$	Amino acid	241	151.95	1.908	241	73.9	-19	-14	-17	-19	-29	-15
Cytidine	$C_9H_{13}N_3O_5$	Amino acid	244.1	112.05	6.393	244.1	95	-19	-13	-23	-19	-42	-19
Cytidine 3',5'-cyclic monophosphate	$C_9H_{12}N_3O_7P$	Nucleoside	306	112.1	4.093			-11	-22	-22			
Cytidine monophosphate	$C_9H_{14}N_3O_8P$	Nucleotide	324	112.05	2.26	324	95	-26	-14	-23	-26	-54	-19
Cytosine	$C_4H_5N_3O$	Nucleotide	112	95.1	4.044			-30	-23	-20			
Dimethylglycine	$C_4H_9NO_2$	Amino acid	104.1	58.05	2.189	104.1	44.05	-12	-16	-11	-12	-38	-17
Dopa	$C_9H_{11}NO_4$	Amino acid	198.1	152.1	6.278			-30	-14	-11			
Dopamine	$C_8H_{11}NO_2$	Amino acid derivative	154.1	91.05	8.078	154.1	137.05	-13	-27	-19	-13	-15	-15
Epinephrine	$C_9H_{13}NO_3$	Catecholamine	184.1	166.1	7.164	184.1	77	-15	-12	-19	-15	-44	-15
FAD	$C_{27}H_{33}P_2N_9O_{15}$	Catecholamine	786.15	136.1	6.213	786.15	348.1	-32	-47	-28	-32	-23	-26
FMN	$C_{17}H_{21}N_4O_9P$	Coenzyme	455	97	6.193	455	78.9	24	28	18	24	38	14

Fumaric acid	$C_4H_4O_4$	Coenzyme	115	71.1	4.571	115	26.95	12	11	12	12	14	26
Glutamic acid	$C_5H_9NO_4$	Organic acid	147.9	84.1	2.253	147.9	56.1	-11	-17	-17	-11	-30	-23
Glutamine	$C_5H_{10}N_2O_3$	Amino acid	147.1	84.15	2.073	147.1	130.1	-11	-18	-17	-11	-16	-27
Glutathione	$C_{10}H_{17}N_3O_6S$	Amino acid	308	179.1	4.543			-25	-13	-13			
Glycine	$C_2H_5NO_2$	Peptide	75.9	30.15	2.029			-17	-11	-30			
Guanine	$C_5H_5N_5O$	Amino acid	150	133	5.623	150	66.1	17	19		17	30	
Guanosine	$C_{10}H_{13}N_5O_5$	Purine base	284	152	6.187	284	135	-22	-12	-17	-22	-39	-15
Guanosine 3',5'-cyclic monophosphate	$C_{10}H_{12}N_5O_7P$	Nucleoside	346	152.05	5.393	346	135.05	-28	-22	-17	-28	-48	-27
Guanosine monophosphate	$C_{10}H_{14}N_5O_8P$	Nucleotide	364	152.05	2.552	364	135	-30	-17	-17	-30	-49	-27
Histamine	$C_5H_9N_3$	Nucleotide	112.1	95.05	5.803	112.1	41.05	-30	-17	-20	-30	-29	-17
Histidine	$C_6H_9N_3O_2$	Amino acid derivative	155.9	110.1	2.901	155.9	56.1	-18	-15	-23	-18	-35	-22
Homocysteine	$C_4H_9NO_2S$	Amino acid	136	90.1	3.188	136	56.1	-10	-13	-18	-10	-22	-21
Homocystine	$C_8H_{16}N_2O_4S_2$	Amino acid	3	1	269	136.05	4.321	-21	-11	-15	-21	-34	-19
Hypoxanthine	$C_5H_4N_4O$	Amino acid	137	55.05	4.251	137	110	-10	-32	-22	-10	-22	-23

Inosine	$C_{10}H_{12}N_4O_5$	Purine derivative	269.1	137.05	6.211	269.1	118.95	-23	-10	-15	-23	-41	-24
Isocitric acid	$C_6H_8O_7$	Nucleoside	191.2	111.1	2.358	191.2	73	12	15	20	12	24	27
Isoleucine	$C_6H_{13}NO_2$	Organic acid	132.1	86.2	7.241	132.1	69.15	-30	-12	-17	-30	-19	-14
Kynurenine	$C_{10}H_{12}N_2O_3$	Amino acid	209.1	192.05	8.34	209.1	94.1	-18	-11	-22	-18	-14	-19
Lactic acid	$C_3H_6O_3$	Amino acid derivative	89.3	89.05	2.795			10	7	17			
Leucine	$C_6H_{13}NO_2$	Organic acid	132.1	86.05	7.52	132.1	30.05	-30	-12	-17	-30	-18	-29
Lysine	$C_6H_{14}N_2O_2$	Amino acid	147.1	84.1	2.894			-11	-18	-18			
Malic acid	$C_4H_6O_5$	Amino acid	133.1	114.95	2.358	133.1	71.15	18	17	24	18	17	26
Methionine	$C_5H_{11}NO_2S$	Organic acid	149.9	56.1	5.304	149.9	104.1	-11	-18	-11	-11	-14	-21
Methionine sulfone	$C_5H_{11}NO_4S$	Amino acid	180	79.2	2.184			19	15	14			
Methionine sulfoxide	$C_5H_{11}NO_3S$	Amino acid	166	74.1	2.206	166	55.95	-12	-14	-15	-12	-25	-22
NAD	$C_{21}H_{27}N_7O_{14}P_2$	Coenzyme	663.1	541.05	3.882	663.1	540.1	26	17	38	26	17	26
Niacinamide	$C_6H_6N_2O$	Vitamin	123.1	80.05	5.344	123.1	53.1	-10	-23	-16	-10	-31	-21
Nicotinic acid	$C_6H_5NO_2$	Organic acid	124.05	80.05	4.08	124.05	78.05	-17	-22	-15	-17	-24	-15
Norepinephrine	$C_8H_{11}NO_3$	Catechol amine	170.1	152.15	4.988	170.1	107.1	-14	-10	-17	-14	-21	-22

Ophthalmic acid	$C_{11}H_{19}N_3O_6$	Organic acid	290.1	58.1	5.35	290.1	161.1	-24	-23	-23	-24	-13	-18
Ornitine	$C_5H_{12}N_2O_2$	Amino acid	133.1	70.1	2.679	133.1	116.05	-10	-18	-14	-10	-15	-24
Orotic acid	$C_5H_4N_2O_4$	Organic acid	155	111.1	2.588	155	42.1	17	13	22	17	22	15
Oxidized glutathione	$C_{20}H_{32}N_6O_{12}S_2$	Peptide	611.1	306	6.253	611.1	143.05	24	24	20	24	48	28
Pantothenic acid	$C_9H_{17}NO_5$	Organic acid	220.1	90.15	6.249	220.1	72.05	-18	-15	-18	-18	-23	-14
Phenylalanine	$C_9H_{11}NO_2$	Amino acid	166.1	120.1	8.068	166.1	103.1	-12	-15	-24	-12	-29	-20
Proline	$C_5H_9NO_2$	Amino acid	116.1	70.15	2.609	116.1	28.05	-30	-18	-14	-30	-35	-30
Pyruvic acid	$C_3H_4O_3$	Organic acid	86.9	87.05	2.585	86.9	42.95	12	7	16	12	12	15
S-Adenosylhomocysteine	$C_{14}H_{20}N_6O_5S$	Amino acid derivative	385.1	134	8.197	385.1	136.05	-15	-21	-15	-15	-21	-29
S-Adenosylmethionine	$C_{15}H_{22}N_6O_5S$	Amino acid derivative	399.1	250.05	6.939	399.1	136.1	-15	-16	-18	-15	-30	-29
Serine	$C_3H_7NO_3$	Amino acid	105.9	60.1	1.96			-25	-12	-11			
Serotonin	$C_{10}H_{12}N_2O$	Amino acid derivative	177.1	160.1	10.527	177.1	77.05	-15	-13	-18	-15	-49	-14
Succinic acid	$C_4H_6O_4$	Organic acid	117.3	73	4.055	117.3	99.05	13	13	28	13	14	19

Symmetric dimethylarginine	$C_8H_{18}N_4O_2$	Amino acid	203.1	70.15	6.817	203.1	71.1	-17	-27	-13	-17	-27	-14
Taurocholic acid	$C_{26}H_{45}NO_7S$	Organic acid	514.2	107.1	7.781	514.2	124.05	30	55	20	30	55	25
Threonine	$C_4H_9NO_3$	Amino acid	120.1	74.15	2.133	120.1	56.05	-27	-13	-14	-27	-17	-11
Thymidine	$C_{10}H_{14}N_2O_5$	Nucleoside	243.1	127.1	6.175			-18	-12	-14			
Thymidine monophosphate	$C_{10}H_{13}N_2O_8P_2$	Nucleotide	322.9	81.1	3.07	322.9	207.1	-25	-22	-16	-25	-9	-15
Thymine	$C_5H_6N_2O_2$	Pyrimidine base	127.1	54.05	5.448	127.1	110.05	-11	-29	-23	-11	-8	-21
Tryptophan	$C_{11}H_{12}N_2O_2$	Amino acid	205.1	188.15	10.092	205.1	146.1	-16	-12	-23	-16	-18	-17
Tyrosine	$C_9H_{11}NO_3$	Amino acid	182.1	136.1	6.694	182.1	91.1	-14	-15	-27	-14	-30	-18
Uracil	$C_4H_4N_2O_2$	Nucleoside	113	70	2.986			-20	-17	-13			
Uric acid	$C_5H_4N_4O_3$	Organic acid	167.1	123.95	3.159	167.1	96.2	12	19	24	12	19	17
Uridine	$C_9H_{12}N_2O_6$	Nucleoside	245	113.05	4.444			-19	-10	-23			
Valine	$C_9H_{12}N_2O_6$	Amino acid	118.1	72.15	4.761	118.1	55.05	-27	-13	-15	-27	-24	-11
Xanthine	$C_5H_4N_4O_2$	Purine base	151	108	4.093	151	42	17	20	20	17	21	15

Table S5. Parameters of Compound Discoverer workflow.

Workflow Node	Workflow Parameter	Workflow Information
Input files		.raw data
Select Spectra	Spectrum Properties Filter	Lower RT limit: 0<br>Upper RT limit: 0
	Scan Event Filters	Polarity Mode: Is +
Align Retention Times	General Settings	Alignment Model: Adaptive curve<br>Maximum Shift [min]: 0.3<br>Mass Tolerance: 2 ppm
Detect Compounds	General Settings	Mass Tolerance: 2 ppm<br>Intensity Tolerance [%]: 30<br>S/N Threshold: 5<br>Min. Peak Intensity: 500 000<br>Ions: [2M + FA + H]-1; [2M + H]+1; [2M + K]+1; [2M + Na]+1; [2M - H]-1; [M + 2H]+2; [M + Cl]-1; [M + FA - H]-1; [M + H]+1; [M + H + K]+2; 2M + H + MeOH]+1; [M + H + Na]+2; [M + H – H_2O]+1; [M + K]+1; [M + Na]+1; [M - 2H]-2; [M + 2H + K]-1; [M -H]-1; [M -H_2O]-1<br>Min. Element Counts: C H<br>Max. Element Counts: C90 H190 Br3 Cl4 K2 N10 Na2 O15 P5 S5
Group Compounds	Compound Consolidation	Mass Tolerance: 2 ppm<br>RT Tolerance [min]: 0.2
	Fragment Data Selection	Preferred Ions: [M - H] +1; [M- H] -1
Search mzCloud	General Settings	Compound Classes: Endogenous Metabolites, Excipients/ Additives/ Colourants, Extractables/ Leachables, Natural Products/ Medicines, Natural Toxins, Personal Care Products/ Cosmetics, Small Molecule Chemicals, Steroids/ Vitamins/ Hormones, Therapeutic/ Prescription Drugs<br>Precursor Mass Tolerance: 10 ppm<br>FT Fragment Mass Tolerance: 10 ppm<br>Library: Autoprocessed, Reference<br>Post. Processing: Recalibrated<br>Annotation Matching. Fragments: True
	DDA Search	Identity Search: Cosine<br>Match Activation Type: True<br>Match Activation Energy: Match with Tolerance<br>Activation Energy: 20

		Apply Intensity Threshold: True<br>Similarity Search: None<br>Match Factor Threshold: 60
	DIA Search	Use DIA Scans: False<br>Max. Isolation Width [Da]: 500<br>Match Activation Type: False<br>Match Activation Energy: Any<br>Activation Energy Tolerance: 100<br>Apply Intensity Threshold: False<br>Match Factor Threshold: 20
Predict Compositions	Prediction Settings	Mass Tolerance: 2 ppm<br>Min. Element Counts: C. H<br>Max. Element Counts: C90 H190 Br3 Cl4 K2 N10 Na2 O15 P5 S5<br>Min. RDBE: 0<br>Max. RDBE: 40<br>Min. H/C: 0.1<br>Max. H/C: 4<br>Max.# Candidates: 10<br>Max.# Internal Candidates: 200
	Pattern Matching	Intensity Tolerance [%]: 30<br>Intensity Threshold [%]: 0.1<br>S/N Threshold: 3<br>Min. Spectral Fit [%]: 30<br>Min. Pattern Cov. [%]: 90<br>Use Dynamic Recalibration: True
	Fragments Matching	Use Fragments: True<br>Mass Tolerance: 2 ppm<br>S/N Threshold: 5
Map to Metabolika Pathways	Search Settings	Metabolika pathways: All<br>Search Mode: By Formula or Mass
	By Mass Search Settings	Mass Tolerance: 2 ppm
	By Formula Search Settings	Max. # of Predicted Compositions to be searched per Compound: 3
	Display Settings	Max. # of Pathways in 'Pathways' column: 20
Apply mzLogic	General Settings	FT Fragment Mass Tolerance: 10 ppm<br>IT Fragment Mass Tolerance: 0.4 Da<br>Max. # Compounds: 0<br>Max. # mzCloud Similarity Results to consider per Compound: 10<br>Match Factor Threshold: 30
Assign Compound Annotations	General Settings	Mass Tolerance: 2 ppm
	Data Sources	Data source #1: mzCloud Search<br>Data source #2: Predicted Compositions<br>Data source #3: MassList Search

		Data source #4: ChemSpider Search<br>Data source #5: Metabolika Search
	Sorting Rules	Use mzLogic: True<br>Use Spectral Distance: True<br>SFit Threshold: 20<br>SFit Range: 20
Fill Gaps	General Settings	Mass Tolerance: 2 ppm<br>S/N Threshold: 5<br>Use Real Peak Detection: True
Apply QC Correction	General Settings	Min. QC Coverage [%]: 30<br>Max. QC Area. RSD [%]: 30<br>Max. Corrected QC Area RSD [%]: 25<br>Max. # Files Between QC Files: 15
Mark Background Compounds	General Settings	Max. Sample/ Blank: 5<br>Max. Blank/ Sample: 0<br>Hide Background: True
Differential Analysis	General Settings	Log10 Transform Values: True

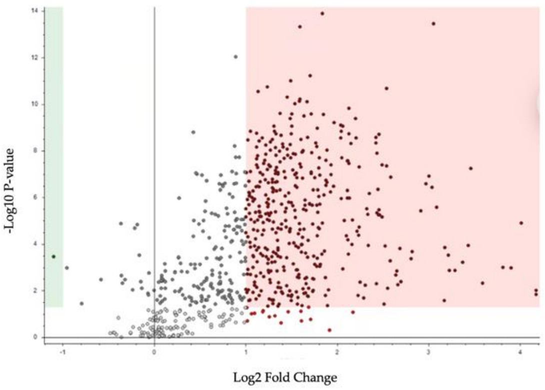

Figure S1. Untargeted differential analysis of sample weight showing volcano plot of altered metabolites, plotted as log2 fold change vs -log10P. Metabolites that are significantly increased in 50 mg samples compared to 20 mg samples are highlighted in red and those that are significantly decreased are shown in green. Differences in metabolite level were defined by a log2 fold change of 1 and the significance level was set at $p < 0.05$.

(a)

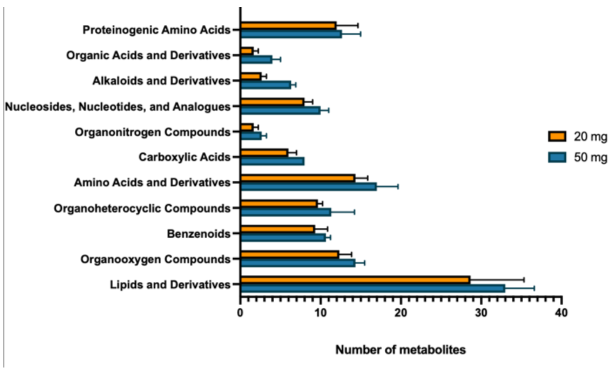

(b)

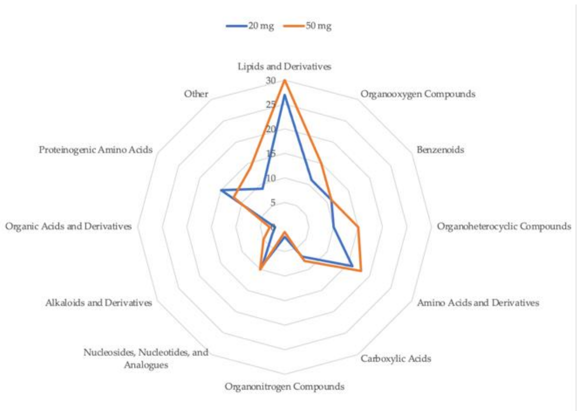

Figure S2. Untargeted metabolite class analysis of sample weight. (a) Comparison of the total number of metabolites identified by chemical class in 20 mg and 50 mg samples (n=3), performed in triplicate. (b) Radar plot comparing the relative abundance of metabolite classes in 20 mg and 50 mg samples. Data were expressed as mean ± SEM and statistical significance was assessed using unpaired t-test.

(a)

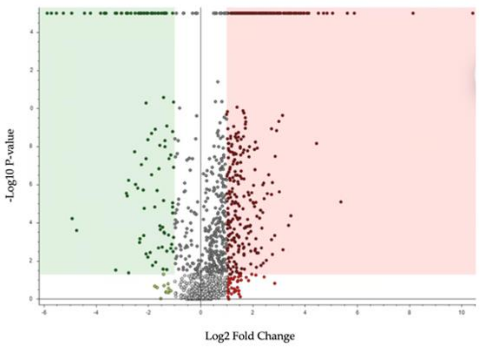

(b)

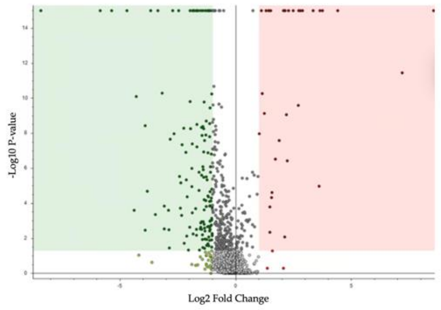

(c)

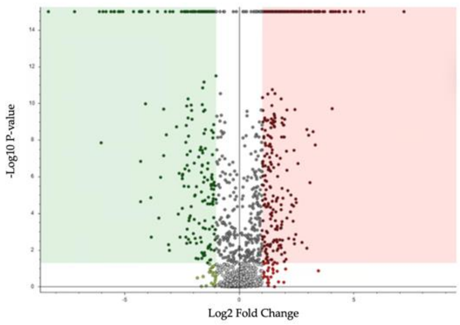

Figure S3. Untargeted differential analysis of extraction solvent showing volcano plot of altered metabolites between (a) MeOH vs. MeOH/ H_2O, (b) $CHCl_3$/ MeOH vs. MeOH/ H_2O, and (c) $CHCl_3$/ MeOH vs MeOH, plotted as log2 fold change vs -log10P. Metabolites that are significantly increased are highlighted in red and those that are significantly decreased are shown in green. Differences in metabolite level were defined by a log2 fold change of 1 and the significance level was set at $p < 0.05$.

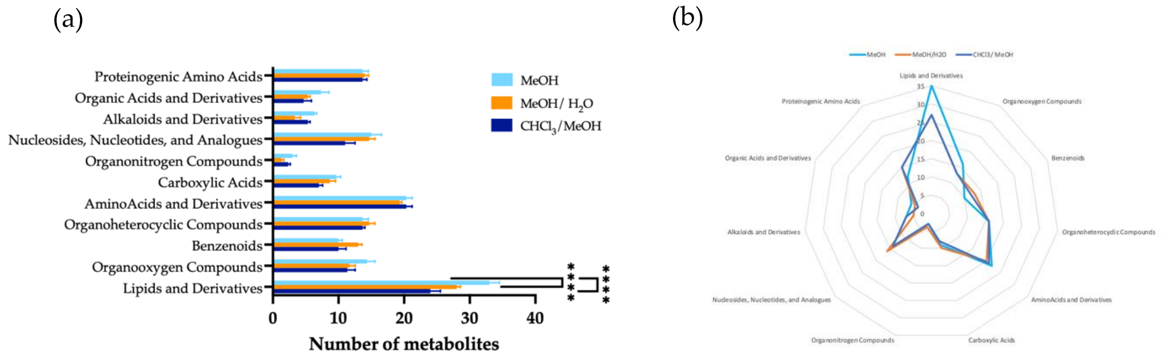

Figure S4. Untargeted metabolite class analysis of extraction solvent. (a) Comparison of the total number of metabolites identified by chemical class in samples extracted with MeOH, MeOH/H_2O, and $CHCl_3$/ MeOH (n=3), performed in triplicate. (b) Radar plot comparing the relative abundance of metabolite classes in samples extracted with MeOH/ H_2O, MeOH, and $CHCl_3$/ MeOH. Data were expressed as mean ± SEM and statistical significance was assessed using a one-way ANOVA. *$p < 0.05$, **** $p < 0.0001$.

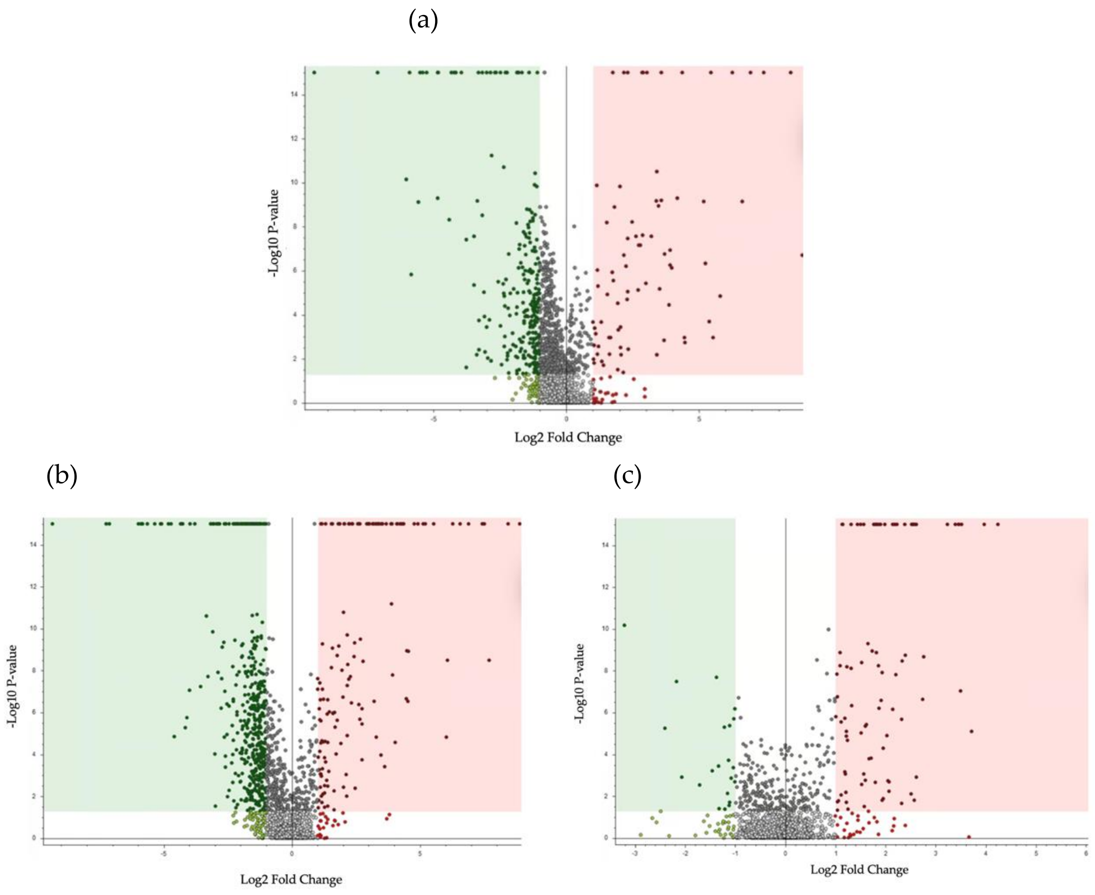

Figure S5. Untargeted differential analysis of extraction solvent showing the volcano plot of altered metabolites between (a) sonication vs. bead beating, (b) freeze-thaw vs. bead beating and (c) freeze-thaw vs. sonication, plotted as log2 fold change vs -log10P. Metabolites that are significantly increased are highlighted in red and those that are significantly decreased are shown in green. Differences in metabolite levels were defined by a log2 fold change of 1 and the significance level was set at $p < 0.05$.

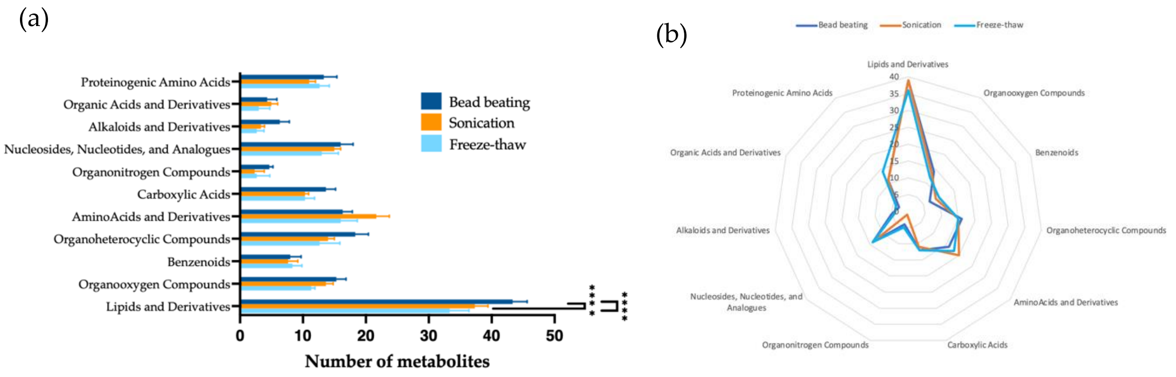

Figure S6. Untargeted metabolite class analysis of cellular disruption method. (a) Comparison of the total number of metabolites identified by chemical class in samples extracted using bead beating, sonication, and freeze-thaw cycles (n=3), performed in triplicate. (b) Radar plot comparing the relative abundance of metabolite classes in samples extracted using bead beating, sonication, and freeze-thaw cycles. Data are expressed as mean ± SEM and statistical significance was assessed using a one-way ANOVA, **** $p < 0.0001$.

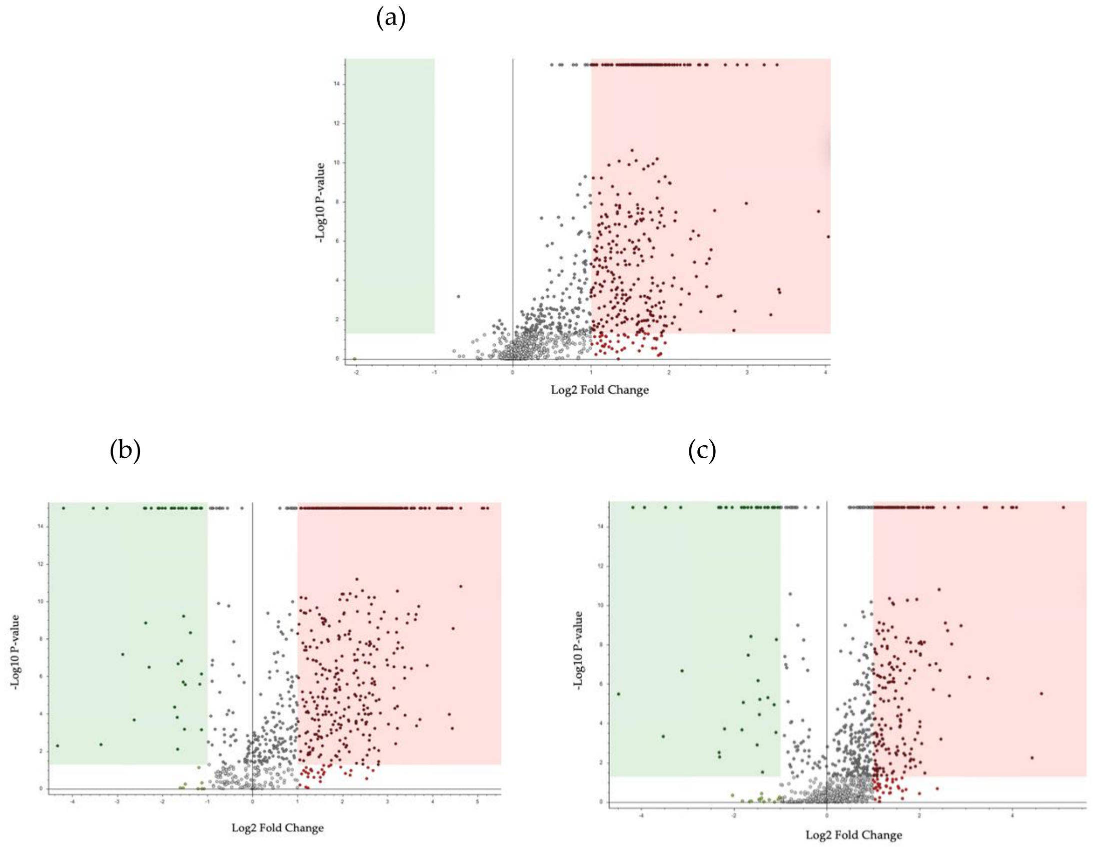

Figure S7. Untargeted differential analysis of sample-solvent ratio showing volcano plot of altered metabolites between (a) 1:10 vs. 1:5, (b) 1:20 vs. 1:5 and (c) 1:20: vs. 1:10, plotted as log2 fold change vs -log10P. Metabolites that are significantly increased are highlighted red and those that are significantly decreased are shown in green. Differences in metabolite level were defined by a log2 fold change of 1 and the significance level was set at $p < 0.05$.

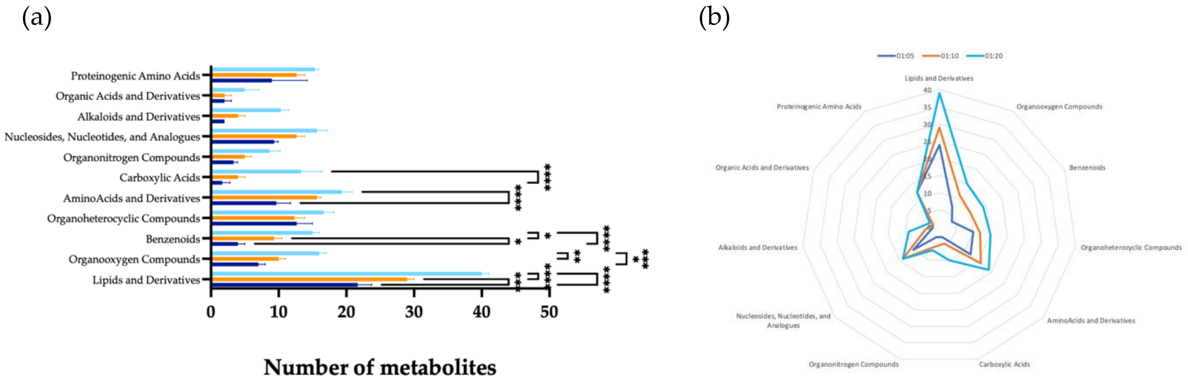

Figure S8. Untargeted metabolite class analysis of sample-to-solvent ratio. (a) Comparison of the total number of metabolites identified by chemical class in samples extracted using sample-to-solvent ratios of 1:5, 1:10, 1:20 (n=3), performed in triplicate. (b) Radar plot comparing the relative abundance of metabolite classes in samples extracted using 1:5, 1:10, 1:20. Data were expressed as mean ± SEM and statistical significance was assessed using a one-way ANOVA., ** $p < 0.01$, **** $p < 0.0001$.

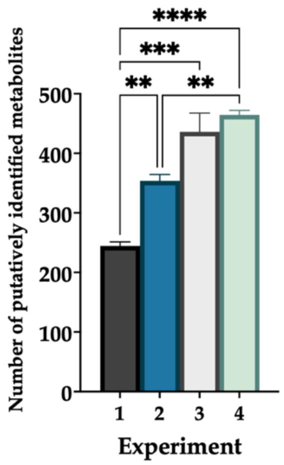

Figure S9. Comparison of individual optimization experiments. Total number of putatively identified metabolites given by optimal parameters of each experiment. Experiment 1, Analysis of Extraction Weight; Experiment 2, Analysis of Extraction Solvent; Experiment 3; Analysis of Cellular Disruption Method; Experiment 4, Analysis of Sample-to-Solvent Ratio. Data were expressed as mean ± SEM and statistical significance was assessed using a one-way ANOVA. **$p < 0.01$, *** $p < 0.001$, **** $p < 0.0001$.

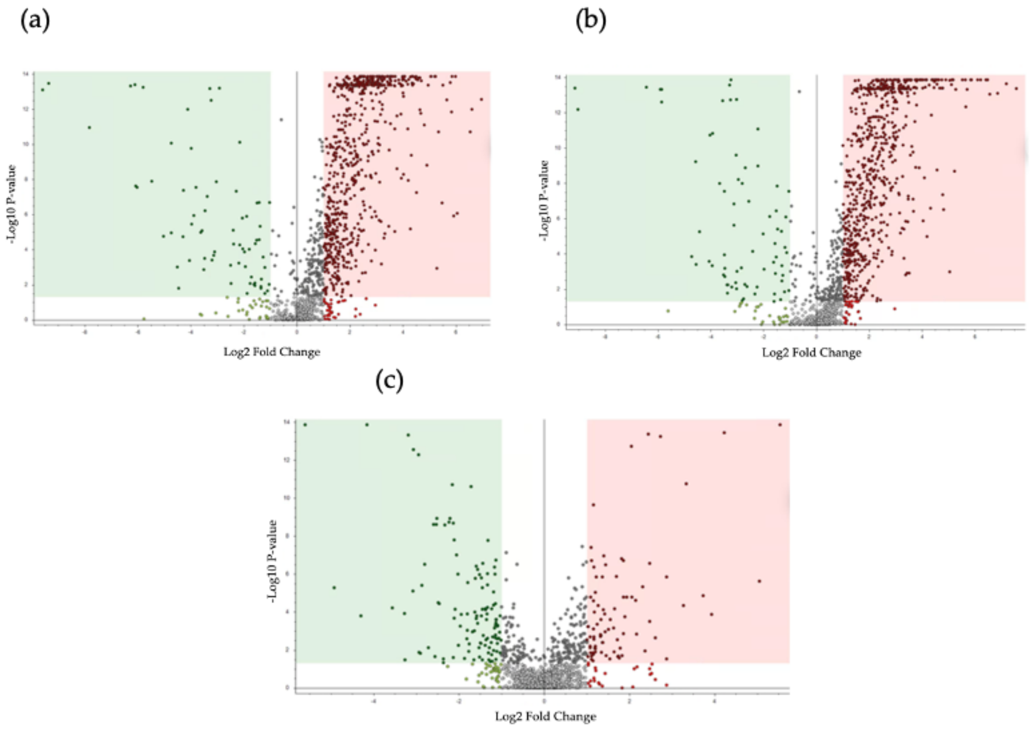

Figure S10. Untargeted differential analysis of cell lysis techniques. Volcano plot of (a) HC vs. CD; (b) CoD vs. CD (c) HC vs. CoD, for all patients. Log2 fold change vs. -log10P. Metabolites. Metabolites that are significantly increased are highlighted in red and those that are significantly decreased are shown in green. Differences in metabolite level were defined by a log2 fold change of 1 and the significance level was set at $p < 0.05$.

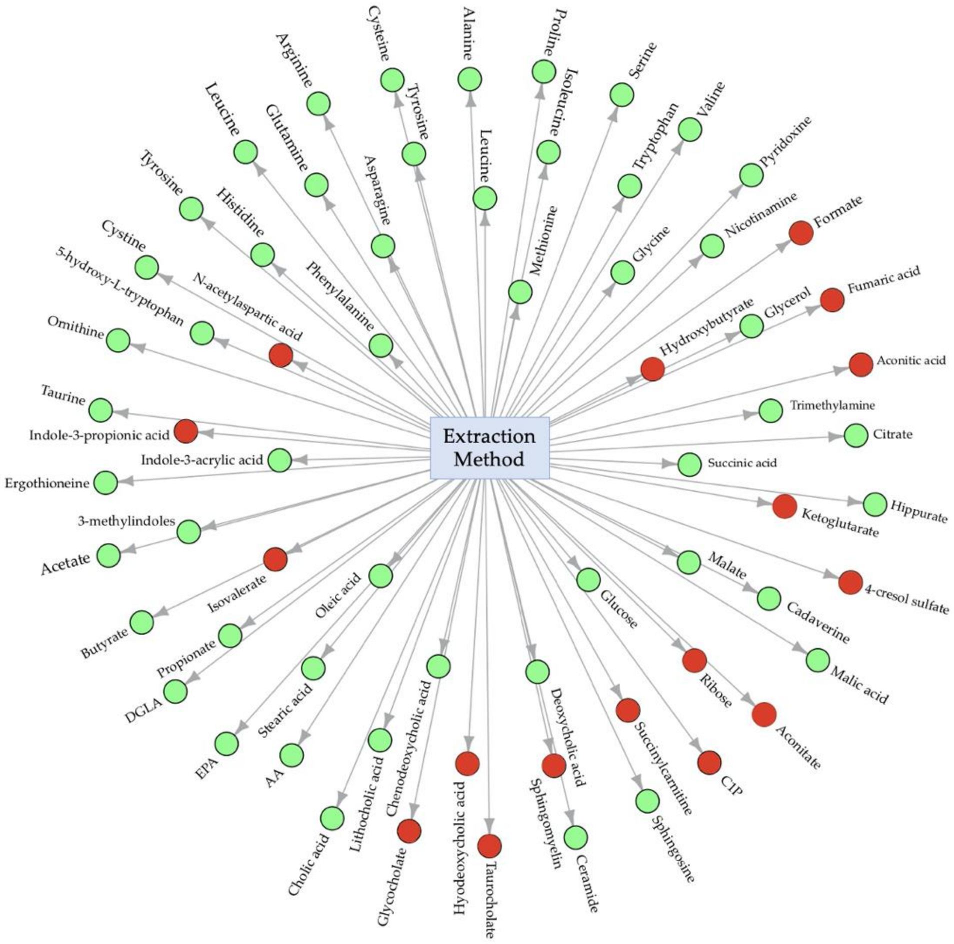

Figure S11. Central network analysis of developed metabolite extraction method. Circles shown in green represent metabolites successfully extracted using the developed method and circles shown in red represent metabolites not found using the developed method. C1P, Ceramide-1-phosphate; AA, Arachidonic acid; EPA, Eicosapentanenoic acid; DGLA, Dihomo-gamma linolenic acid.

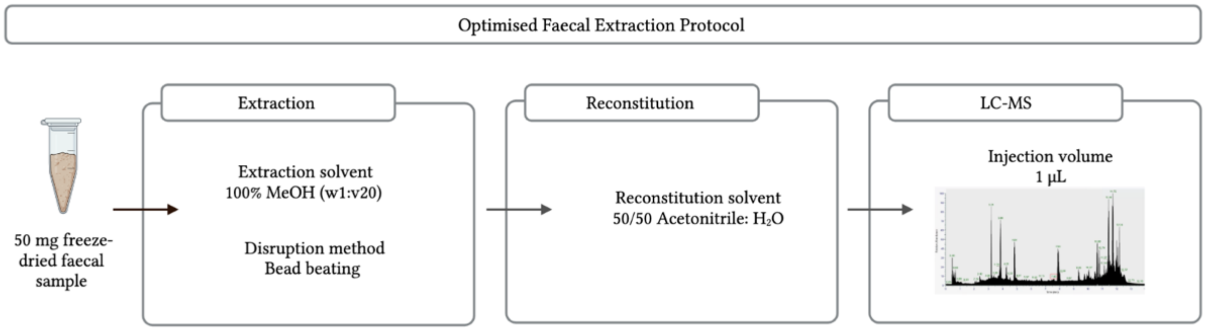

Figure S12. Summary of the developed methodology pipeline. Multi-parameter analysis showed that 50 mg samples give the strongest MS output, and from the extraction solvents analysed, MeOH is the most effective. Additionally, cellular metabolite release is optimal using bead beating as the cell lysis method. Combining optimised parameters provides an experimental protocol for faecal metabolite extraction that can be used for metabolomic analysis.
